# Mupirocin in the Treatment of Staphylococcal Infections in Chronic Rhinosinusitis: A Meta-Analysis

**DOI:** 10.1371/journal.pone.0167369

**Published:** 2016-12-01

**Authors:** Jong Seung Kim, Sam Hyun Kwon

**Affiliations:** 1 Department of Otolaryngology-Head and Neck Surgery, College of Medicine, Chonbuk National University, Jeonju, Republic of Korea; 2 Research Institute of Clinical Medicine of Chonbuk National University–Biomedical Research Institute of Chonbuk National University Hospital, Jeonju, Republic of Korea; University of South Australia, AUSTRALIA

## Abstract

**Background:**

Saline irrigation of the nasal cavity is a classic and effective treatment for acute or chronic rhinosinusitis. Topical antibiotics such as mupirocin have been widely used for recalcitrant chronic rhinosinusitis. Therefore, the purpose of this study was to evaluate the effect of saline irrigation using mupirocin.

**Methods:**

A systematic literature review and meta-analysis of mupirocin saline irrigation were performed using EMBASE, MEDLINE, and Cochrane library through December 2015. Data were analyzed with R 3.2.2 software. A random effects model was used because of the diversity of included studies. Sensitivity analysis of particular tested groups and single proportion tests were also performed. The main outcome measure was residual staphylococcal infection, as confirmed by culture or PCR.

**Results:**

Two RCTs, two prospective studies and two retrospective studies were included. A random effects model meta-analysis of the pooled data identified a relative risk of residual infection of 0.13 (95% CI: 0.06–0.26, p<0.05) with low heterogeneity (I^2^ = 0%). The proportion of residual staphylococcal infections after 1 month was 0.08 (95% CI: 0.04–0.16). However, this proportion increased to 0.53 at 6 months (95% CI: 0.27–0.78).

**Conclusions:**

The short-term use of mupirocin has a strongly reductive effect on staphylococcal infection in chronic rhinosinusitis. Although there is currently a lack of clear evidence, future studies with well-designed inclusion criteria and randomized controlled trials are needed to examine mupirocin’s long-term effect on chronic rhinosinusitis.

## Introduction

Saline irrigation of the nasal cavity is a classic and powerful method to treat acute or chronic rhinosinusitis (CRS) [1]. It can be used in allergic or nonallergic rhinitis, postoperative care of endoscopic sinus surgery or septal surgery, and atrophic rhinitis [2,3]. Nasal irrigation works by mechanically removing inflammatory mucin and therefore enhancing mucociliary function [4]. Bacterial biofilms may develop in refractory CRS, which persist after sinus surgery or culture-based antibiotic therapy [5]. However, classic saline irrigation and oral antibiotics have a limited effect on these refractory cases.

Recently, saline irrigation mixed with a topical agent have been introduced to treat these recalcitrant CRS cases [5,6]. Typical topical agents include topical antifungal agents, steroids, surfactants, xylitol and topical antibiotics [7–11]. Of these agents, mupirocin also has significant anti-staphylococcal activity. It is highly effective against methicillin-susceptible (MSSA) and -resistant *Staphylococcus aureus* (MRSA) strains that colonize the nasal cavities and sinuses [6]. In this study, our purpose was to evaluate the efficacy of saline irrigation with mupirocin to treat recalcitrant CRS using a systematic review and meta-analysis.

## Materials and Methods

### Ethical considerations

This is a systematic retrospective review of previously published articles, and no patient identifiable details are included.

### Literature search

Institutional review board approval and patient consent were not required due to the nature of this study. The study was conducted in compliance with the PRISMA check list (Preferred Reporting Items for Systematic Reviews and Meta-Analyses) [12] (**S1 PRISMA Checklist).** The MEDLINE, EMBASE and Cochrane databases were searched for eligible studies published up to and including December 2015. The research key words included “mupirocin,” “irrigation,” “saline,” “sinus surgery,” and “sinusitis.” The following search formula was used in MEDLINE: ("mupirocin"[MeSH Terms] OR "mupirocin"[All Fields]) AND (("sinusitis"[MeSH Terms] OR "sinusitis"[All Fields]) OR (("paranasal sinuses"[MeSH Terms] OR ("paranasal"[All Fields] AND "sinuses"[All Fields]) OR "paranasal sinuses"[All Fields] OR "sinus"[All Fields]) AND ("surgery"[Subheading] OR "surgery"[All Fields] OR "surgical procedures, operative"[MeSH Terms] OR ("surgical"[All Fields] AND "procedures"[All Fields] AND "operative"[All Fields]) OR "operative surgical procedures"[All Fields] OR "surgery"[All Fields] OR "general surgery"[MeSH Terms] OR ("general"[All Fields] AND "surgery"[All Fields]) OR "general surgery"[All Fields])) OR ("therapeutic irrigation"[MeSH Terms] OR ("therapeutic"[All Fields] AND "irrigation"[All Fields]) OR "therapeutic irrigation"[All Fields] OR "irrigation"[All Fields]))

Similar search words were used in each database.

### Selection criteria

Eligible studies met the following inclusion criteria: (1) full-length original article providing dichotomous data to evaluate the effects of mupirocin irrigation on the recurrence of sinusitis; (2) prospective or retrospective cohort study, randomized controlled trial; (3) primary outcomes were expressed with residual staphylococcal colonization which was assessed by culture or polymerase chain reaction (PCR). Studies were excluded if: (1) the treatment modalities contained other topical agents; (2) the article was not written in English; (3) the study had no relation to sinusitis; (4) the study included in vitro studies; (5) the study had duplicate data or incomplete data for calculating the effect sizes; (6) the study was an unpublished trial.

### Data extraction

Two authors independently extracted information from all eligible studies. Any disparities were resolved by consensus. Data including the first author’s name and publication date were collected. The Newcastle-Ottawa Scale was used to assess study quality of case–control and cohort studies, and the Cochrane risk of bias tool was employed for randomized controlled trials (RCTs) [13].

### Treatment outcomes

The proportion of treatment failure cases in the experimental group was obtained by dividing the number of cases with treatment failure by the total number of cases in the study. The proportion of treatment failure cases in the control group was calculated using the same method. The primary outcome was presented in the event of treatment failure which was defined in our study by residual staphylococcal colonization [14–19].

### Statistical analysis

The effect size was represented by the risk ratio of residual staphylococcal infection, which was compared between the mupirocin group and the control group. The standard error was also calculated for each clinical outcome measure. Hedge’s g and standard error were determined for each treatment outcome measure. The 95% confidence interval (CI) was then computed for each type of therapy. The random effects model was used considering the effects from different locations, populations, and heterogenous research groups, which were the main causes of the within-study and between-study variations.

R 3.2.2 software (R Foundation) was used to analyze and graphically display the meta-analysis data. We calculated relative risk with a 95% CI for the treatment outcome. Heterogeneity between studies was assessed using the I^2^ statistic. P-values<0.10 and I^2^>50% indicated evidence of heterogeneity [20]. Potential publication bias was investigated using funnel plots. If a publication bias was suspected, Duval’s trim and fill method was used to correct for the bias using R 3.1.2 [21–23]. A sensitivity analysis was carried out to identify any outlier studies.

## Results

### Study selection

**Fig 1:**
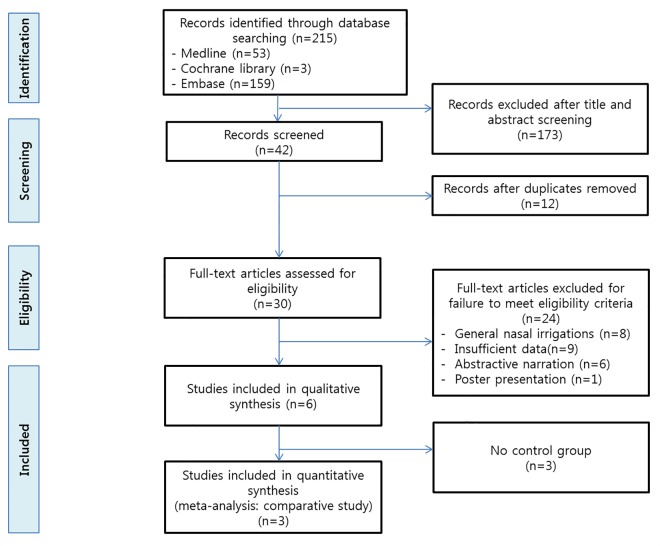
PRISMA guidelines for the study literature search (n = number of studies).

The literature search identified 215 articles. Only six of these articles met the inclusion criteria after the reviewers’ selection. The PRISMA flow diagram of this systematic review is shown in Fig 1. (S1 File) During the screening process of titles and abstracts, a total of 173 studies were excluded because they did not meet the inclusion criteria. Twelve duplicated records were also excluded. The remaining 30 articles qualified for full-text reading, and these were systematically reviewed. After reviewing the full text, 24 publications were excluded because they failed to meet our eligibility criteria (eight articles did not include mupirocin irrigation, nine had insufficient data, six had abstractive narration, and one was a poster presentation). Therefore, six articles were finally included in our qualitative analysis (Table 1). Of these six studies, three studies had no control group. Therefore, three articles were used for effect comparison.

**Table 1:** Demographics of included studies

Study	Mupirocin group		Control group		Concentration of mupirocin	FU	Treatment outcome	Methodology	Quality Score
	Residual Staphlylococcal cases	Total	Residual Staphlylococcal cases	Total					
Jervis-Bardy et al. (2012) [18]	1	9	13	13	125 mg mupirocin/ 240 ml saline for 1 month	1 month	*S*. *aureus* culture, Endoscopy, Sx score	RCT	Low risk†
	1	6				6 months			
Doebbeling et al. (1994) [19]	4	31	30	32	2% mupirocin twice daily for 5 days	1 month	*S*. *aureus* culture	RCT	Unclear risk†
	15	31	23	32		6 months			
	16	31	24	32		1 year			
Seiberling et al. (2013) [16]	1	16	15	16	60 mg mupirocin/ 240 ml saline given once only	10 days	PCR of *S*. *aureus*	Prospective study	8*
Jervis-Bardy and Wormald (2012) [17]	42	57			0.05% mupirocin twice daily for 4 weeks	5 months (mean)	*S*. *aureus* culture	Retrospective chart review	6*
Uren et al. (2008) [14]	1	16			0.05%, 100 mg mupirocin/ 200 ml saline twice daily for 3 weeks	3 weeks	*S*. *aureus* culture, Endoscopy, Sx score	Prospective study	6*
Solares et al. (2006) [15]	1	42			2% mupirocin 440 mg/ 1 L saline twice daily for 4 weeks	4 weeks	*S*. *aureus* culture	Retrospective chart review	6*

Abbreviations: FU, follow-up; RCT, randomized controlled trial; CT, computed tomography; PCR, polymerase chain reaction; Sx, symptom

Risk of bias:

†Cochrane risk of bias

*Newcastle-Ottawa quality assessment scale.

### Meta-analysis

**Fig 2:**
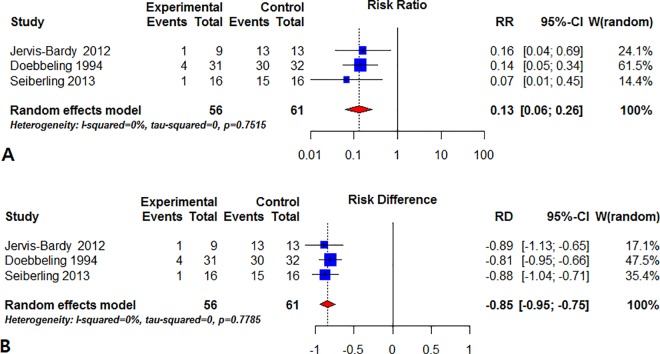
Forest plot of the effect of mupirocin on recalcitrant CRS. (A) The pooled relative risk (RR) of 0.13 was calculated using the inverse variance method and random effects model. (B) The pooled risk difference was calculated to be 0.83.

Six studies were included in the overall analysis. Only three of these studies had a control group and these were analyzed by comparative meta-analysis (Table 1) [16,18,19]. A random effects model of the pooled data from these three studies (n = 101 patients) showed a 0.13 relative risk (RR) of residual infection at 1 month after treatment (95% CI: 0.06–0.26, p<0.01) (Fig 2A) with low heterogeneity (I^2^ = 0%). Mupirocin reduced the risk of residual infection by approximately 87% compared to that in the control group. The pooled risk difference was calculated to be -0.85 (95% CI: -0.75–-0.95, p<0.01) (Fig 2B).

### Evaluation of publication bias

**Fig 3:**
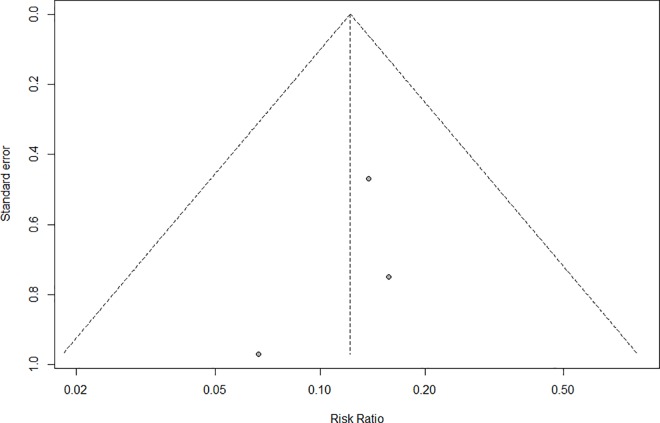
Funnel plot showing that the studies were mostly spread around the midline of the pyramid.

In a funnel plot of mupirocin saline irrigations, the studies were distributed in the center of the plot, suggesting minimal publication bias (Fig 3).

### Sensitivity analysis

**Fig 4:**
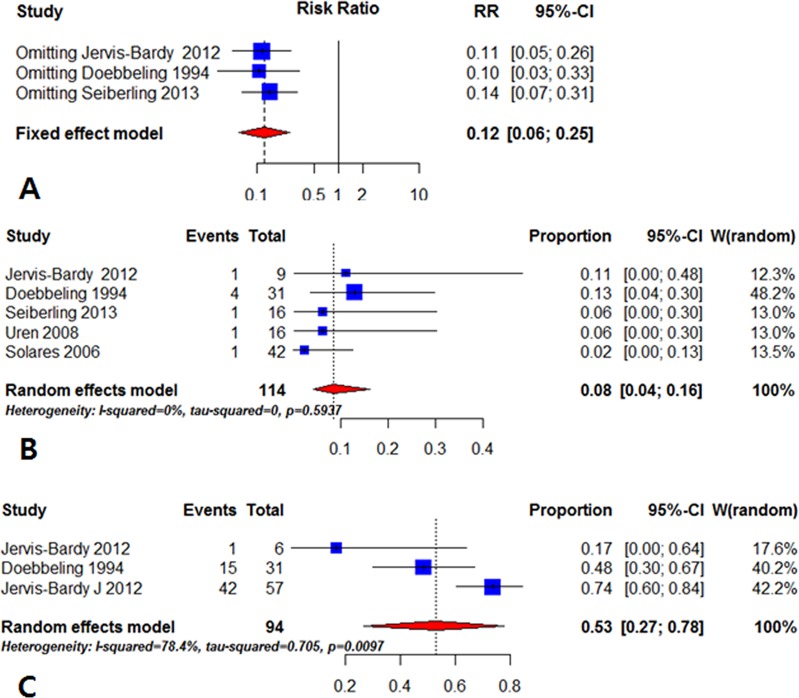
(A) Sensitivity analysis. The pooled risk ratio in the overall comparison was not significantly changed, indicating a stable outcome. (B) Single proportion analysis at 1 month. The proportion of residual *Staphylococcus aureus* was 0.08 (95% CI: 0.04–0.16). (C) The proportion increased to 0.53 at 6 months (95% CI: 0.27–0.78).

A sensitivity analysis was performed to evaluate the stability of the results by removing each study one by one in random order. In the overall comparison, the pooled risk ratio and the stratified analyses were not significantly changed, indicating a stable and robust outcome (Fig 4A).

### Single proportion analysis

Due to limited data from comparison groups (n = 3), single proportion analysis was conducted at 1 and 6 months after mupirocin treatment through all of the included studies [24–27]. After the first month, the proportion of residual staphylococcal infection was 0.08 (95% CI: 0.04–0.16). The proportion increased to 0.53 at 6 months (95% CI: 0.27–0.78) (Fig 4B and 4C).

## Discussion

*Staphylococcus aureus* (*S*. *aureus*) is a common pathogen in chronic rhinosinusitis (CRS) [28]. There are two main theories for the development of recurrent CRS: biofilm formation and superantigen formation. Methicillin-resistant *S*. *aureus* (MRSA) has appeared as a result of β lactamase activity; unfortunately, the prevalence of MRSA is now increasing worldwide [29]. The pathophysiology of biofilm development in CRS includes both bacterial and host factors. The essential organism in a biofilm, which is also associated with poor clinical outcomes, is coagulase-positive *S*. *aureus* [30]. *S*. *aureus* that colonizes within epithelial cells releases enterotoxins and induces a topical multiclonal IgE-formation as well as a steroid-insensitive eosinophilic inflammation [31]. These enterotoxins acts as superantigens. The superantigens induce secretion of interleukin-5, eosinophil cationic protein, and immunoglobulin E, which play a pivotal role in the pathogenesis of CRS with nasal polyps [32,33]. From these mechanisms, therapeutic approaches including antibiotics and anti-interleukin-5 are in the limelight in the nonsurgical treatment of CRS. Topical antibiotics are used clinically for many sites, including the external and middle ears, eyes, oral mucosa, and skin. Topical antibiotics are effective because a high concentration of the drug can be applied locally, with minimal systemic effects. Mupirocin, which is isolated from *Pseudomonas fluorescens*, inhibits bacterial growth [34]. It is unstable in human serum; however, it survives in human nasal secretions, where it retains its anti-staphylococcal activity [35]. Mupirocin is a treatment option for recalcitrant CRS. Although there are reports of mupirocin-resistant *S*. *aureus*, mupirocin is the only effective topical agent against MRSA [36].

Two RCTs and one prospective cohort study were included in our final comparative meta-analysis. All three studies used mupirocin before 1 month and evaluated the effect in the same period [16,18,19]. We found that mupirocin treatment had a risk ratio of 0.13 (95% CI: 0.06–0.26, p<0.05) with low heterogeneity (I^2^ = 0%; p = 0.75). Therefore, mupirocin saline irrigation reduced the risk of residual staphylococcal infection in recalcitrant CRS by approximately 87% at 1 month. Differences in interventions, programs or populations across the studies were the basis for a random effects model; however, there was little heterogeneity [37,38]. The sensitivity analysis did not identify any outlier studies 1 month after mupirocin treatment. Although some studies had a low score quality, this finding supports the view that the risk ratio is robust and stable [20]. After 1 month, we were unable to perform comparative analysis due to insufficient data.

We also conducted single proportion tests to complement the small sample size of the studies. Although proportion analysis provides weaker evidence than randomized controlled trials, it can be applied to systematic reviews yielding proportion data and confidence intervals [39]. The studies by Jervis-Bardy, Solares, Uren and colleagues [14,15,17] were added to the existing three studies (Fig 4B and 4C). A random effects model was used. The proportion of residual staphylococcal infections after 1 month was 0.08 (95% CI: 0.04–0.16). However, this proportion increased to 0.53 at 6 months (95% CI: 0.27–0.78). Doebbeling et al. addressed the long-term effects of mupirocin [19]. This group found that, after 5 days of treatment, mupirocin has a strong effect for 1 month; however, this effect decreases after 6 months and is ineffective at 1 year. Jervis-Bardy et al. also reported that 73.7% of patients subsequently cultured *S*. *aureus* following mupirocin treatment during a mean 5-month follow-up [17]. They postulated that any intracellular or interstitial surviving bacteria may regenerate following subtotal eradication. The increase in residual infections during this interval can also be explained by a recent study reporting that the same staphylococcal strain reappeared rather than colonization by a new strain [40]. Intra-mucosal residence during the culture-negative period is proposed as the probable mechanism.

Traditionally, topical mupirocin has been known for its staphylococcal decolonization effect. Edmundson et al. reported that mupirocin was effective in reducing staphylococcal colonization and it fell from 5.88% to 2.71% on subsequent screening [41]. They reported that all asymptomatic staphylococcal nasal carriers receiving single topical mupirocin were successfully cleared of colonization; however, some required more than one course of treatment. McConeghy et al. reported that topical mupirocin is the standard of care for decolonization of *Staphylococcus*, and it is applied to the anterior nares 2–3 times/day for 5 days [42]. According to their report, since staphylococcal colonization often precedes infection, and infection is associated with significant morbidity and mortality, there is great importance in preventing the transmission of *Staphylococcus* and decolonizing patients who harbor these bacteria. Similarly, we found that topical mupirocin irrigation reduced the risk of residual staphylococcal infection in recalcitrant CRS by approximately 87% at 1 month. *Staphylococcus* that shows antimicrobial resistance is known to be an increasingly important cause of chronic rhinosinusitis [43]. In this regard, mupirocin irrigation has the short-term effect of eradicating residual staphylococcal infection which may aggravate chronic rhinosinusitis.

The content of mupirocin varied across the studies (0.05–2%) [14,15,17,19]. However, the concentration of mupirocin saline was consistent (440–500 mg/L saline) [14,15,18]. Although Seiberling et al. used a low concentration of mupirocin for irrigation, the risk ratio in their study (0.06) was not different from the overall risk ratio (0.13). Only Doebbeling et al. used pure mupirocin ointment, and the others (Jervis-Bardy, Uren, Solares, Seiberling and colleagues) used mupirocin saline washes (rinses) for irrigation. However, our sensitivity analysis showed that omitting Doebbeling’s study did not change the overall risk ratio (RR = 0.10). So the concentration and method of administering mupirocin did not influence its effect on residual staphylococcal infection.

Our study had several limitations. First, our meta-analysis included some studies which had sparse data with regard to long-term follow-up and usage manuals. Further studies that handle these data will enable meta-regression analysis or moderator analysis to be performed. In addition, our study also included a small number of RCTs. It also included observational studies which had low quality scores. Further RCT studies will enrich and substantiate our findings.

## Conclusion

To our knowledge, this is the first meta-analysis to assess the effects of mupirocin saline irrigation on staphylococcal infection in chronic recalcitrant rhinosinusitis. Mupirocin saline irrigation is an effective short-term treatment for recalcitrant staphylococcal CRS. Future studies that address the long-term effects and moderator variables of mupirocin treatment will overcome the present limitations, and contribute additional clinical information.

## Supporting Information

S1 PRISMA Checklist PRISMA 2009 checklist for this study. (DOC)

S1 File PRISMA 2009 flow diagram for this study. (DOC)
