# Supplementary material for: Mupirocin in the Treatment of Staphylococcal Infections in Chronic Rhinosinusitis: A Meta-Analysis
Source: PLoS One. 2016 Dec 1;11(12):e0167369. doi: 10.1371/journal.pone.0167369 (PMC5132234; doi:10.1371/journal.pone.0167369)
Supplement: S1 File — (DOC) [file pone.0167369.s002.doc]

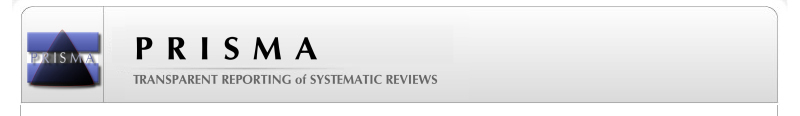
**PRISMA 2009 Flow Diagram**

**Screening**

**Included**

**Eligibility**

**Identification**

Records identified through database searching
(n = 215 )

- Medline (n=53)

- Cochrane library (n=3)

- Embase (n=159)

Additional records identified through other sources
(n = 0 )

Records after duplicates removed
(n = 12 )

Records screened
(n = 203 )

Records excluded
(n = 173 )

- title and abstract screening

Full-text articles assessed for eligibility
(n = 30 )

Full-text articles excluded, with reasons
(n = 24 )

- General nasal irrigations (n=8)
- Insufficient data(n=9)
- Abstractive narration (n=6)
- Poster presentation (n=1)

Studies included in qualitative synthesis
(n = 6 )

Studies included in quantitative synthesis (meta-analysis)
(n = 6 )
